# Additive vs. Subtractive Manufacturing of Zirconia: Influence on Surface Properties, Cell Viability, and *Streptococcus mutans* Adhesion †

**DOI:** 10.3390/jfb17040162

**Published:** 2026-04-01

**Authors:** Ülkü Tuğba Kalyoncuoğlu, Nurten Baysal, Gulcin Akca, Simel Ayyıldız, Burak Yilmaz

**Affiliations:** 1 Department of Prosthodontics, Gülhane Faculty of Dentistry, University of Health Sciences Turkey, Ankara 06018, Turkey; ulkutugbaterzi@gmail.com (Ü.T.K.); simelayyildiz@gmail.com (S.A.); 2 Department of Medical Microbiology, Faculty of Dentistry, Gazi University, Ankara 06490, Turkey; gulcin68@yahoo.com; 3 Medical Design Manufacturing Center, University of Health Sciences Turkey, Ankara 06018, Turkey; 4 Laboratory for Digital Dental Technologies, Department of Reconstructive Dentistry and Gerodontology, School of Dental Medicine, University of Bern, 3012 Bern, Switzerland; 5 Department of Restorative Preventive, and Pediatric Dentistry, School of Dental Medicine, University of Dental Medicine, 3010 Bern, Switzerland; 6 Department of Prosthodontics, Faculty of Dentistry, Ankara University, Ankara 06560, Turkey

**Keywords:** zirconia, cell viability, *Streptococcus mutans*, 3D printing

## Abstract

The surface characteristics of zirconia may influence both soft tissue response and bacterial colonization. This study evaluated the surface roughness and water contact angle of zirconia fabricated by additive manufacturing (material jetting, NPJ) and subtractive manufacturing (milling), and investigated human gingival fibroblast (HGF-1) viability and *Streptococcus mutans* (*S. mutans*) (ATCC 25175) adherence on these surfaces, as well as the possible correlation between roughness and bacterial adhesion. Sixty-four zirconia specimens (1 × 1 × 0.1 cm) were fabricated (*n* = 32 per group), sintered, and standardized by abrasive polishing. Surface roughness and contact angle were measured. Cell viability was assessed using an MTT assay at 24, 48, and 72 h. Bacterial adhesion was quantified after 24 and 48 h of incubation. Data were analyzed using two-way ANOVA, independent *t*-tests, and Pearson correlation (α = 0.05). No significant differences in HGF-1 viability were observed at 24 and 48 h; however, at 72 h, subtractively manufactured zirconia demonstrated higher cell viability than additively manufactured specimens (*p* < 0.001). *S. mutans* adhesion was significantly greater on additively manufactured zirconia at 24 h (*p* = 0.002), with no significant difference at 48 h. Manufacturing technique influenced surface properties and early bacterial adhesion. Both materials exhibited acceptable biocompatibility within the tested conditions.

## 1. Introduction

Ceramics are commonly used in dentistry because of their excellent intraoral biocompatibility and ability to mimic natural teeth with satisfactory mechanical and optical properties [1]. Among the various types of ceramics, zirconia has been popular for complete crowns or for fixed partial dentures (FPDs) [2,3,4], and as an implant or abutment material for implant-retained restorations [5,6].

One of the benefits of conventional zirconia is its biocompatibility; no harmful substances that might cause adverse reactions in the body are released, which is crucial for the long-term health of the tissues surrounding the implant–abutment complex and restoration margins [7]. The characteristics of zirconia surfaces, including surface charge and energy, roughness, stiffness, texture, and wettability, have the potential to influence bacterial adherence [8]. These factors are crucial in maintaining the permanence of the barrier, limiting or inhibiting the risk of inflammation at the gingival and bone level, or keeping microorganisms from infecting the transmucosal and bone area [8,9]. Despite the presence of various microorganisms, it is well established that *S. mutans*, a well-known cariogenic bacterium, is one of the primary colonizers of dental plaque in the oral cavity [10,11,12,13]. *S. mutans* biofilm accumulation on tooth surfaces triggers inflammatory responses, which may lead to early-stage soft tissue diseases such as gingivitis or peri-implant mucositis [14,15,16]. Gingivitis is the mildest form of periodontal disease and is primarily caused by bacterial biofilm accumulation at the gingival margin [14,15,16]. Bacterial biofilm accumulation at the gingival margin is also the most common cause of periodontal disease. Therefore, examining the adhesion behavior of *S. mutans*, which plays a central role in the development of dental caries, can offer valuable insights into biofilm formation and its implications for clinical caries progression [17,18].

Dental zirconia structures are typically fabricated by subtractive manufacturing (SM) methods based on CAD-CAM technologies by using sintered or pre-sintered materials. In recent years, additive manufacturing (AM) techniques have also become increasingly popular for zirconia fabrication. In addition to the advantages of SM, AM techniques can create complex shapes directly and produce multiple customized components in a time-efficient manner, while minimizing raw material waste [1,2,19]. Stereolothography (SLA) [20,21] and digital light processing (DLP) [22,23] technologies have been in use for AM of zirconia, and more recently, NanoParticle Jetting (NPJ) has been introduced to manufacture zirconia restorations [19,24,25,26,27].

Unlike SLA and DLP, NPJ technology uses a binder-based material jetting process, which can result in zirconia properties that may differ from those manufactured with SLA or DLP; surface chemistry and microtopography of zirconia may vary, which can critically affect biological responses, such as bacterial adhesion and cellular interactions. In this respect, each AM technique should be evaluated independently for its properties and clinical impact, as the printing principles in each are different. To date, research studies have focused mainly on the physical and mechanical properties of zirconia produced with NPJ [19,24,25,26,27,28].

As mentioned in a previous study, AM zirconia manufactured with NPJ demonstrated mechanical properties similar to SM zirconia and was reported as adequate for dental applications [19]. Surface and biological investigations on AM zirconia, however, have been performed mainly when SLA and DLP techniques were used [20,21,22,23] and NPJ technology’s biological effects remain largely unexplored; prior studies have not addressed NPJ zirconia’s interaction with bacteria and HGF-1 cells, and none have investigated *S. mutans* adhesion and cell viability. The present study was designed to fill this gap by providing the first comprehensive evaluation of cytocompatibility and *S. mutans* adhesion on NPJ zirconia, aiming to evaluate the surface roughness, wettability, and HGF-1 cell viability at 24, 48, and 72 h on both AM and SM zirconia surfaces, as well as the adherence capacity of *S. mutans* to these materials. The present study also aims to investigate whether a correlation between surface roughness (SR) and *S. mutans* adherence exists. It was hypothesized that the manufacturing type would affect the surface roughness, wettability, HGF-1 cell viability, and *S. mutans* adherence capacity of zirconia. It was also hypothesized that a correlation would be found between the SR and *S. mutans* adherence on zirconia.

## 2. Materials and Methods

### 2.1. Sample Preparation and Surface Treatments

Zirconia specimens were produced using either 3D printing, also known as AM technology or SM. The specimens were designed in the dimensions of 1 × 1 × 0.1 cm^3^ using a software program (3-Matic, Materialise, Leuven, Belgium). As described in a previous study [19], the AM specimens were manufactured using a material jetting (MJ) printer based on patented NPJ technology (XJet, Carmel the 1400, Rehovot, Israel). Additively manufactured zirconia specimens were fabricated using NanoParticle Jetting (NPJ) technology (Carmel 1400, XJet Ltd., Rehovot, Israel). In this process, zirconia nanoparticles are dispersed in a liquid ink and deposited layer-by-layer to form the printed structure. According to the manufacturer’s technical documentation [29] and previous reports [28], the printing ink consists of zirconia nanoparticles suspended in an organic solvent with dispersing agents and additives. During subsequent thermal debinding and sintering stages, the organic components are removed, resulting in a dense zirconia ceramic structure. Following AM, the specimens were subjected to debinding and sintering in a furnace, increasing the temperature from 25 °C to 100 °C at a rate of 1 °C/min for complete drying, then holding at 100 °C for 60 min. Afterwards, the temperature was increased to 500 °C at a rate of 1 °C/min and held for 1 min. Then, the temperature was raised to 950 °C at a rate of 4 °C/min and held for 1 min, and subsequently raised to 1450 °C at a rate of 1 °C/min and held for 180 min. Finally, the temperature was reduced to room temperature at a rate of 4 °C/min. Specimens in the SM group were produced by using a semi-sintered yttria-stabilized ZrO2 (Nacera Pearl; Doceram, Dortmund, Germany) and a milling machine (Yenadent D43; Yenadent, Istanbul, Turkey), followed by sintering at 1500 °C in a furnace (Tegra MP1500; Teknik Dental, Istanbul, Turkey). A total of 64 square-shaped specimens were prepared for experimental groups (*n* = 32). The sample size for this study, which aimed to evaluate surface roughness and wettability measurements, was calculated using G*Power software (version 3.1.9.7), based on an a priori power analysis with a significance level of α = 0.05, a statistical power of 88%, and an effect size of 0.80 (G* power statistical program; v.3.1.9.7). The effect size was selected in line with Cohen’s conventions for a large effect size in experimental studies [30] and previous studies focusing on similar topics [18,31]. One surface of each specimen was ground using silicon carbide (SiC) and Al_2_O_3_ abrasive paper with grit sizes ranging from #600 to 1200 (Waterproof Paper; Atlas Zımpara, İstanbul, Turkey). After this surface treatment, the specimens were cleaned in an ultrasonic bath for 15 min each in 70% ethanol and distilled water.

### 2.2. Surface Characterization

The surface roughness (SR) of each specimen was subsequently measured using a contact profilometer (TR100 Surface Roughness Tester; Checkline Europe BV, Cologne, Germany), following previously established protocols [13,19]. The cut-off length (λc) was set to 0.25 mm. Three measurements were obtained from different locations of each specimen, and the mean value was calculated. The average roughness (Ra) values were obtained from the profilometry measurements. A contact angle measurement device (Krüss DSA-30, Hamburg, Germany) equipped with an image analyzer and a video camera was used to assess the WCA of each specimen (*n* = 32). WCA degree values were acquired for each zirconia specimen by applying a droplet of distilled water (2 µL) onto the surfaces. The WCA was determined by calculating the average of 3 measurements of the angle of the tangent to the surface of the liquid droplet in various locations on the surface of the specimen.

Two specimens were then randomly selected from each group and coated with a sputter coater (JFC-1500, JEOL Ltd., Tokyo, Japan) with an approximate coating thickness of 10 nm to examine the surface examination and chemical composition by using SEM. Surface morphology was examined using a field-emission scanning electron microscope (FE-SEM) (SU-7000, Hitachi, Tokyo, Japan) operated at an accelerating voltage of 15 kV and at magnifications of ×500 and ×5000. Elemental composition was analyzed using an energy-dispersive X-ray spectroscopy (EDS) detector (Ultim Max, Oxford Instruments, Abingdon, UK).

### 2.3. Cell Culture and Cell Viability Testing

The specimens from each group (*n* = 30) were autoclaved using a standard glassware process at 121 °C for one hour after the SR and WCA measurements. The normal human gingival fibroblast cell line HGF-1 (ATCC CRL-2014) was used in the present study. The cells were cultured in Dulbecco’s modified Eagle media supplemented with 10% fetal calf serum, 100 U/mL penicillin, and 100 g/mL streptomycin. To eliminate the senescent cells, freshly grown cells from the fourth to fifth passage were used. Then, all specimens were seeded with HGF-1 cells in 24-well plates, adding 1 mL of full growth media. Each well had a seeding density of 5 × 10^4^ cells/mL. The cells were grown in a 5% (*v*/*v*) CO_2_ incubator (MCO-18AIC, Sanyo Co., Osaka, Japan) at 37 °C. Grown cells were assessed by using the 3-(4,5-dimethylthiazol-2-yl)-2,5-diphenyl-tetrazolium salt (MTT) assay (5 mg/mL; Sigma, St. Louis, MO, USA) which was prepared by dissolving the crystals of insoluble formazan in 1 mL of isopropanol with 0.04 N HCl. After the incubation process (24–48–72 h) carried out at 37 °C, the amount of resulting colored solution was determined by measuring its absorbance at 570 nm using a multi-well microplate reader. (Bio Tek Epoch, Santa Clara, CA, USA). As a control, culture medium was employed. The experiment was independently carried out in triplicate. The cell viability was determined as a percentage using the following formula [32]:

$$\%\;\mathrm{Cell}\;\mathrm{viability}\;=\;\frac{Sample\;absorbance-blank\;absorbance}{Control\;absorbance-blank\;absorbance}\times 100$$

### 2.4. Streptococcus mutans Adhesion Assay

For *S. mutans* adherence assessment, new specimens (*n* = 6 from each group for each time point) were prepared. The *S. mutans* was employed to evaluate the microbial attachment. *S. mutans* was cultivated for 24–48 h on tryptic soy agar (TSA, Merck, Darmstadt, Germany) in a 37 °C incubator with 5% CO_2_. *S. mutans*’ bacterial suspension was made in tryptic soy broth (TSB, Merck, Darmstadt, Germany) with a concentration of 1.5 × 10^8^ CFU/mL according to the 0.5 McFarland test standard. The measurement was carried out using spectrophotometry, and the result was obtained at an optical density of 0.600 at a wavelength of 450 nanometers (OD450 nm: 0.600). The specimens were placed into each well of a 48-well cell culture plate and contaminated with the bacterial suspension. The plates were then incubated for 24 and 48 h under the same conditions. After completing the chosen incubation periods, the specimens were removed and washed three times with one ml of PBS (pH: 7.0) solution to remove the unattached bacterial cells. Then, they were put in 1.5 mL Eppendorf tubes containing 1 mLof PBS (pH: 7.0) solution, vortexed vigorously for two minutes and sonicated twice for 10 s separately to detach the cells from the surface of the materials [33]. After vortexing for one minute to determine and count the number of adherent bacterial cells to the specimens, they were independently cultured under the same incubation conditions as before. Additionally, to confirm the absence of any remaining bacteria on the surfaces of the materials, samples were retaken from the materials’ surfaces and cultured under the same conditions. The absence of any bacterial growth on the culture media indicated that no bacteria remained adhered to the material surface. The grown colonies were enumerated and calculated as CFU (Colony Forming Unit)/mL.

### 2.5. Statistical Analysis

The statistical analysis was carried out using a software program (IBM SPSS, v25; IBM Corp, Armonk, NY, USA). The normality of the results was assessed using the Shapiro–Wilk test. Levene’s test was used to evaluate the homogeneity of variances. Two-way ANOVA was used to compare the groups and time points, while an independent *t*-test was selected to compare the results between groups. To examine the relationship between bacterial adherence and SR, Pearson correlation analysis was employed (α = 0.05).

## 3. Results

The SR and WCA values are summarized in Table 1.

**Figure 1:**
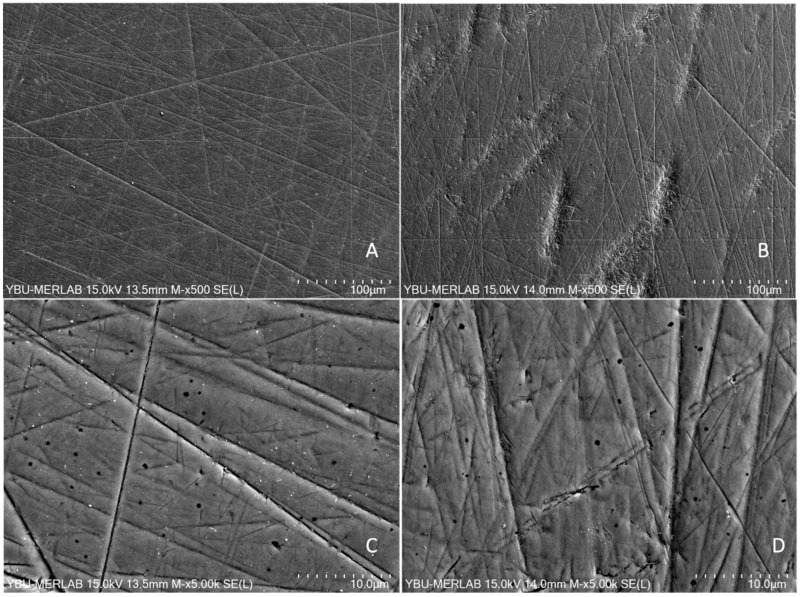
SEM images show top surfaces of the zirconia core materials after grinding. (**A**,**C**) show SM, (**B**,**D**) show the AM groups.

The independent *t*-test revealed a statistically significant difference between the groups for Ra values and WCA (*p* < 0.001). (Table 1). The SEM images of the AM and SM specimens after grinding revealed different surface topography patterns. The AM surfaces appeared more irregular with numerous micro-asperities, whereas the SM surfaces exhibited relatively smoother areas interrupted by linear grinding grooves. (Figure 1).

**Figure 2:**
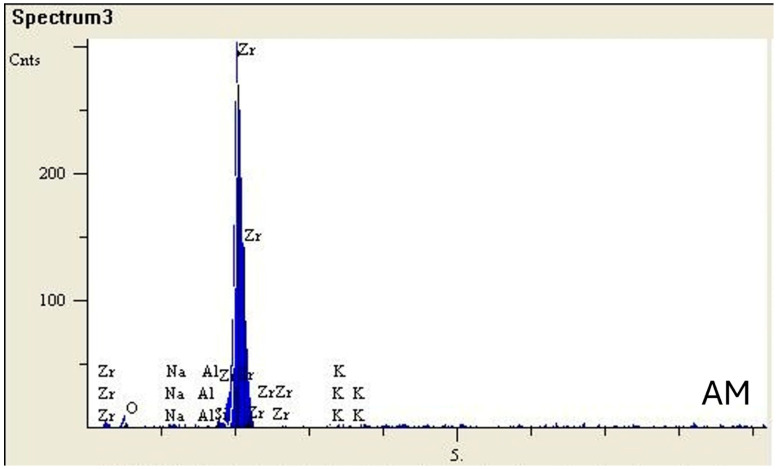

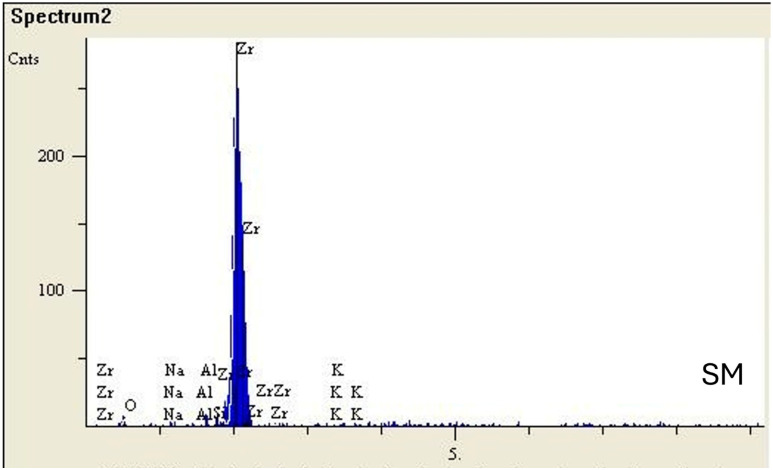
SEM-EDS analysis of the SM and AM groups.

Figure 2 displays the SEM-EDS results for AM and SM specimens. EDS analysis showed similar elemental compositions in both groups, with zirconium and oxygen being the predominant elements. The AM specimens contained approximately 93.4 wt.% Zr and 6.4 wt.% O, along with trace amounts of Na (0.01 wt.%), Al (0.09 wt.%), and K (0.05 wt.%). Similarly, the SM specimens consisted of 92.2 wt.% Zr and 7.1 wt.% O, with minor amounts of Na (0.11 wt.%), Al (0.10 wt.%), and K (0.44 wt.%).

**Figure 3:**
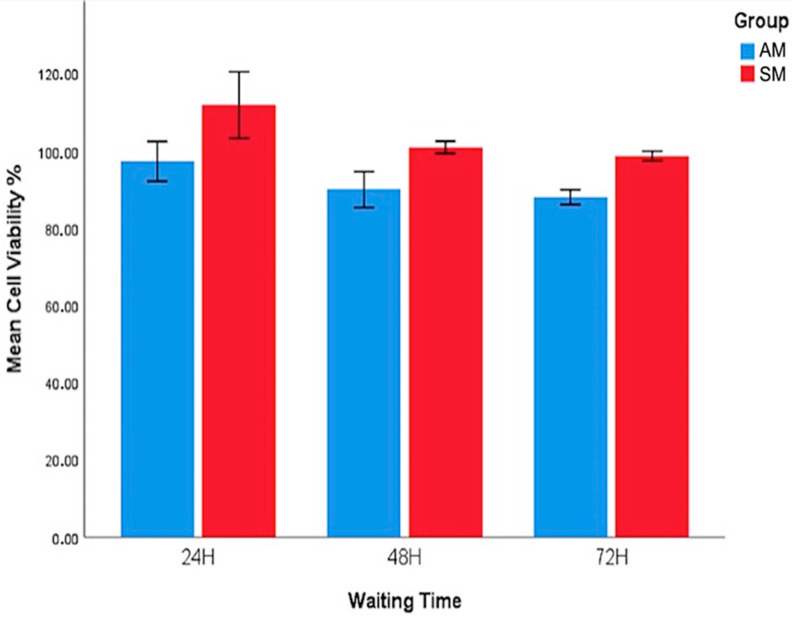
Cell viability of AM and SM zirconia specimens.

The cell viability assessments were defined through an MTT assay, and the results are shown in Table 2 and Figure 3.

No significant differences in viability were found between AM and SM groups at 24 (*p* = 0.176) and 48 h (*p* = 0.052). However, significant differences were shown at 72 h between the groups; SM specimens showed higher cell viability than AM specimens (*p* < 0.005).

**Figure 4:**
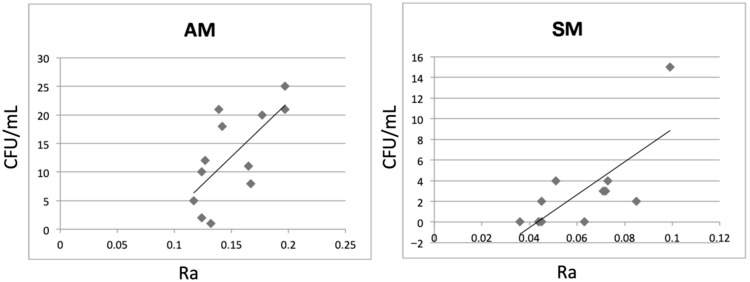
Correlation between surface roughness (Ra) and *S. mutans* adhesion for AM zirconia (r = 0.687) and SM zirconia (r = 0.662).

The bacterial adherence results at 24 h revealed a significant difference between the groups (*p* < 0.005). No statistical difference regarding bacterial adherence was found for 48 h of incubation (*p* = 0.062). There was a statistically significant positive correlation between the SR values of AM zirconia and the amount of *S. mutans* adhesion (r = 0.687, r^2^ = 0.47, *p* < 0.05), indicating that 47% of the variance in bacterial adhesion could be explained by surface roughness. Similarly, a statistically significant positive correlation was observed between the SR values of SM zirconia and the amount of *S. mutans* adhesion (r = 0.662, r^2^ = 0.44, *p* < 0.05), with 44% of the variance explained by surface roughness (Figure 4). These findings suggest a moderate relationship between SR and *S. mutans* adherence for both manufacturing methods.

## 4. Discussion

All hypotheses were accepted as the zirconia manufacturing type affected the SR, wettability, cell viability and *S. mutans* adherence. In addition, a correlation was found between the SR and *S. mutans* adherence.

Biocompatible materials are required to maintain fibroblastic attachment around prosthetic restoration or dental implant transmucosal area for protection against oral microorganisms and to provide long-term health of the tissue and success of the restorations [32,34]. The surface characteristics of materials play an essential role in biological parameters such as cell attachment, proliferation, and functionality [35]. A previous study reported that the average roughness threshold required to maintain the seal between the epithelial cells and the surface had been accepted as Ra: 0.2 µm [31]. Moreover, the water contact angle of a material below 90° is determined as hydrophilic, an essential critical value for fibroblast attachment, proliferation, and soft tissue integration [33]. In the present study, the hydrophilicity of both zirconia surfaces was similar, with contact angle values below 90°, although statistically significant differences were detected between the groups. Surface roughness measurements were performed following the same grinding procedure for all specimens. The Ra value of the AM group (0.138 ± 0.026 µm) was significantly higher than that of the SM group (0.064 ± 0.017 µm). The SEM images supported the surface roughness findings, showing that the surfaces of SM specimens appeared smoother than those of AM specimens (Figure 1A–D). Previous studies revealed that AM NPJ zirconia exhibited lower hardness values (AM: 1116.9 HV1; SM: 1501.4 HV1) [19]. Therefore, it is hypothesized that, due to its lower hardness, the AM specimens may experience greater scratching during grinding with sandpapers compared to a harder SM specimen [18]. Goo et al. reported that during surface grinding or polishing procedures, abrasive particles move across the surface of the zirconia specimen, and these particles possess a hardness greater than that of the material being abraded [36]. Therefore, polishing procedures may induce scratching on materials that are softer than the abraded particles. In the present study, no polishing kit was used for finishing procedures, which can be considered a limitation, because surface polish may play a role in cell adhesion.

Evaluating cell viability is crucial to assessing the biocompatibility of dental materials for their clinical application [37]. In the present study, direct contact cell viability assays were used to assess the cell viability of zirconia specimens. Although no statistically significant differences in cell viability were observed between the SM and AM groups at 24 and 48 h, the AM group showed a significant decrease in viability at 72 h (AM: 88.00 ± 4.65; M: 98.67 ± 2.94; *p* < 0.001). The fact that the *p*-value for 48 h was borderline to being not statistically significant should be interpreted carefully. Nevertheless, the results were evaluated based on the classification proposed by Sjogren et al. [38]. In terms of cytotoxicity, the AM zirconia demonstrated adequate cytocompatibility, as the cell viability values at 24 and 48 h were above 90%, indicating that the material is not cytotoxic. Although the cell viability values for the AM zirconia specimens decreased at 72 h, they remained above 70%, which is consistent with non-cytotoxic behavior [32].

Likewise, cellular compatibility and the success of dental restorations in the long term also depend on the quality and quantity of attached microbial biofilm. *S. mutans* has been identified as one of the primary etiologic agents of initial attachment on the surfaces of restorations [10]. Although the comparison of *S. mutans* adherence assessment within the groups revealed a significant difference at 24 h (AM: 17.67 ± 5.68; M: 4.0 ± 5.62; *p* < 0.005), no statistically significant difference was found at 48 h of incubation (AM: 8 ± 7.38; M: 1.5 ± 1.76; *p* = 0.062). Vo et al. [18] and Yu et al. [31] reported that increased microorganism adhesion was associated with higher SR of ceramic surfaces. The findings of the present study are consistent with these previous reports. Yu et al. indicated that the rougher surfaces of the zirconia specimens increase the contact area for adhesion of *S. mutans* [31]. Branco et al. reported that the body and the surface of AM zirconia exhibited a more porous structure due to the manufacturing technique [1]. Additionally, the finding reported by Ann et al. [39] that SLA-based additively manufactured zirconia exhibits increased *S. mutans* adhesion compared to milled zirconia further supports the importance of manufacturing-related surface characteristics in influencing microbial attachment.

In the present study, it is hypothesized that the porous structures of AM specimens and potential scratching on AM surfaces revealed in SEM images may have contributed to the increased SR, which, in turn, may have enhanced the adhesion of *S. mutans* to AM zirconia during the 24 h incubation period.

Pearson correlation analysis revealed statistically significant positive correlations between SR and *S. mutans* adhesion in both groups (AM: r = 0.687, r^2^ = 0.47; SM: r = 0.662, r^2^ = 0.44; *p* < 0.05). The coefficients of determination indicate that surface roughness accounts for approximately 47% and 44% of the variance in bacterial adhesion for AM and SM groups, respectively, suggesting a moderate relationship. The remaining variance implies that additional surface-related factors may also contribute to bacterial colonization.

Song et al. [40] found that bacterial adherence, the initial step in biofilm formation, is influenced by several factors, including surface charge, roughness, pattern, wettability, chemistry, and stiffness of the material. Additionally, it was stated that bacterial attachment decreases over time due to the detachment of bacteria [40]. These results may help explain the reduced *S. mutans* adherence observed at 48 h incubation for both the SM and AM groups in the present study. The fact that no initial pellicle was formed on surfaces in the present study limits the clinical interpretation of findings; nevertheless, the fact that the methodology was standardized across tested material groups still enables comparisons with previous studies on restorative materials, which did not include pellicle in their methodologies either [10,18,31,41,42]. Frąckiewicz et al. compared biofilm formation by multi-species oral microorganisms on zirconia samples produced using additive (SLA) and milling techniques, and stated that there were statistically insignificant, minimal differences, particularly with regard to *S. mutans* [21]. In their study, biofilm formation was assessed only at 24 h. Although the same time point was evaluated in the present study, a significant difference in *S. mutans* adherence between the groups was detected. This inconsistency could be attributed to differences in the additive manufacturing technologies employed, SLA in the referenced study and nanoparticle jetting (NPJ) in the present study, as well as variations in surface characteristics and qualitative methods used in their study. These methodological distinctions must be considered when analyzing and comparing the outcomes of both studies. To ensure reliable comparisons, such investigations should be conducted under similar laboratory conditions and with standardized experimental protocols.

To the best of the authors’ knowledge, this is the first report in the literature to examine both the cell viability and *S. mutans* adherence to AM zirconia manufactured through the NPJ technique. Therefore, the findings may provide basic initial information on the cytocompatibility of NPJ zirconium and lay the groundwork for future comparative evaluations against AM technologies that have been studied in the literature for longer periods of time, such as SLA. Due to ethical considerations, obtaining detailed information about the composition of this newly manufactured zirconia material is challenging, which presents a limitation to the present study. Comprehensively interpreting the EDX analysis was particularly challenging due to the lack of this confidential information. Nevertheless, EDX analysis indicated no major differences in the chemical composition between the two zirconia groups. This finding warrants further evaluation using alternative measurement techniques. In addition, different zirconia slurries should be tested for their structural, surface, mechanical, and biological properties. Future investigations may also benefit from incorporating more comprehensive surface characterization approaches to further elucidate the relationship between zirconia surface properties and biological interactions. As the process of oral microorganism adhesion is complex and influenced by various factors, further extensive research involving multiple oral species, particularly focusing on multi-species biofilm formation, specimens coated with salivary proteins and mucins, and pellicle formation, should be performed in future studies.

## 5. Conclusions

The manufacturing technique significantly influenced the surface roughness (SR) and wettability of zirconia. Cell viability at 72 h was higher for subtractive manufactured (SM) zirconia compared to additive-manufactured (AM) zirconia; however, both materials demonstrated acceptable biocompatibility. *Streptococcus mutans* adherence at 24 h was greater on AM zirconia, which may be attributed to its increased surface roughness. Despite the higher early bacterial adherence, AM zirconia showed potential as a promising alternative for clinical applications.

Further in vitro and clinical investigations are required, particularly those focusing on multi-species biofilm models and advanced surface characterization techniques, to fully evaluate the long-term clinical performance of AM zirconia in dental restorations.

## Figures and Tables

**Table 1:** Independent *t*-test results for surface roughness (Ra) and water contact angle (WCA) of groups.

	AM		SM		*p Value*
	Mean	Std. Dev.	Mean	Std. Dev.	
Ra	0.138	±0.026	0.064	±0.017	*<0.001*
WCA	65.97	±10.33	53.96	±10.21	*<0.001*

AM: Additive manufacturing; SM: subtractive manufacturing. *p*-values were obtained from independent *t*-test comparing AM and SM groups. Ra; *t*-test = 13.262; *p* < 0.001. WCA; *t*-test = 4.676; *p* < 0.001.

**Table 2:** Two-way ANOVA test results for cell viability at different time points.

Cell Viability %		AM		SM		* p Value
		Mean	Std. Dev.	Mean	Std. Dev.	
Duration	24 h	97.25	±12.51	111.83	±21.11	*0.176*
	48 h	90.00	±11.42	100.83	±3.87	*0.052*
	72 h	88.00	±4.65	98.67	±2.94	*0.001*
** P.		*0.319*		*0.238*		

* Significance levels of the difference between groups at the same time point. ** Significance levels of the difference between time points within the same group.

## Data Availability

The original contributions presented in this study are included in the article. Further inquiries can be directed to the corresponding author.
